# Psychotherapeutic and neurobiological processes associated with ayahuasca: A proposed model and implications for therapeutic use

**DOI:** 10.3389/fnins.2022.879221

**Published:** 2023-01-31

**Authors:** Daniel Perkins, Simon G. D. Ruffell, Kimberley Day, Diego Pinzon Rubiano, Jerome Sarris

**Affiliations:** ^1^School of Population and Global Health, University of Melbourne, Melbourne, VIC, Australia; ^2^School of Social and Political Science, University of Melbourne, Melbourne, VIC, Australia; ^3^Psychae Institute, Melbourne, VIC, Australia; ^4^Centre for Mental Health, Swinburne University, Melbourne, VIC, Australia; ^5^Onaya Science, Iquitos, Peru; ^6^NICM Health Research Institute, Western Sydney University, Sydney, NSW, Australia; ^7^Florey Institute of Neuroscience and Mental Health, Melbourne, VIC, Australia

**Keywords:** ayahuasca, DMT, harmala alkaloids, psychedelic therapy, psychotherapeutic processes, neurobiology, mental health, health behaviors

## Abstract

Ayahuasca is a psychoactive Amazonian plant brew. It is usually made from the *Banisteriopsis caapi* vine (Spruce ex Griseb. Morton, Malpighiaceae), which contains three primary harmala alkaloids, along with the leaves of *Psychotria viridis* (Ruiz et Pavon, Rubiaceae) in which the potent psychedelic dimethyltryptamine (DMT) is found. DMT-harmaloid concoctions have gained popularity in recent years, due to growing anecdotal and scientific reports of therapeutic benefits associated with their consumption. Ayahuasca is now ingested in a variety of different settings across the globe, from traditional ethnobotanical to so called “neo-shamanic” ceremonies. Furthermore, related preparations involving alternative sources of DMT and harmala alkaloids are becoming increasingly common as knowledge of ayahuasca continues to spread internationally. This article reviews the existing literature and draws on original qualitative data from a large cross-sectional study of ayahuasca drinkers, to propose a model of psychotherapeutic processes associated with the consumption of ayahuasca. We assert that it is these processes, facilitated by a range of neurobiological effects, that lead to beneficial mental health and wellbeing outcomes. Our proposed model identifies five key psychotherapeutic processes or effects inherent to the ayahuasca experience; somatic effects; introspection and emotional processing; increased Self-connection; increased spiritual connection, and finally the gaining of insights and new perspectives. We note some important differences in these processes compared with other classic psychedelics as well as the implications of the model for the therapeutic use of ayahuasca. Improved understanding of the psychotherapeutic processes involved with the ayahuasca experience will better equip practitioners to work with this potentially transformative concoction and enable the optimization of therapeutic treatment models for potential clinical use.

## Introduction

A DMT-harmala alkaloid based traditional medicine known as ayahuasca has been used by indigenous cultures in the Amazon basin for healing, spiritual and other purposes for at least hundreds of years (Naranjo, 1986; Shanon, 2002). It is typically made from the ayahuasca vine [*Banisteriopsis caapi (Spruce) Morton, Malpighiaceae*] and the leaves of chacruna (*Psychotria viridis Ruiz et Pavon, Rubiaceae*) or chaliponga [*Diplopterys cabrerana (Cuatrec.) B. Gates, Malpighiaceae*] (Ruffell et al., 2020). The word ayahuasca is from the Quechua language meaning “vine of the souls” (McKenna et al., 1984), and this concentrated liquid decoction can produce powerful changes in awareness and consciousness, which are considered central to its therapeutic effect (Wolff et al., 2019).

In addition to continuing traditional use, ayahuasca has been adopted as a religious sacrament by several Brazilian syncretic religions that have now expanded internationally to Europe, North America, and Australia (Shanon, 2002; Tupper, 2009; Trichter, 2010; Lowell and Adams, 2017). Recent decades have also seen large numbers of international tourists visiting South American countries seeking ayahuasca’s renowned therapeutic and spiritual or personal development effects (Kavenská and Simonová, 2015). At the same time there has been a rapid growth in ceremonies using ayahuasca or related preparations (involving alternative plant sources of DMT and harmala alkaloids) in underground indigenous styled neo-shamanic ceremonies taking place in countries across the world (Tupper, 2009; Trichter, 2010; Gearin, 2015). In this article we use the term ayahuasca to refers to DMT-harmala alkaloid brews using traditional and non-traditional ingredients.

The article draws on existing literature and qualitative data from the Global Ayahuasca Project (GAP) to expound a novel model of the psychotherapeutic processes underlying ayahuasca’s reported mental health and wellbeing benefits. We begin by undertaking a narrative review of current evidence relating to ayahuasca therapeutic effects and proposed neurobiological and psychotherapeutic mechanisms. We then outline our proposed model and discuss its implications for the therapeutic and potential clinical use of ayahuasca.

We hypothesize that the use of ayahuasca specific treatment protocols that are informed by modalities congruent with the processes outlined in our model, will optimize therapeutic outcomes in clinical settings. Our team is currently working toward a Phase 2 clinical trial with an ayahuasca inspired drug, which will utilize a manualized treatment model incorporating such elements for participants with treatment resistant depression and alcohol use disorder.

## Methodology

We use original qualitative data from the GAP study to elucidate the key psychotherapeutic processes that we propose are associated with beneficial mental health and wellbeing effects associated with ayahuasca consumption. Benny Shanon (2002) describes language as *a natural expression of the human cognitive system*, and in a similar fashion to the seminal work of Shanon, *The antipodes of the mind: Charting the phenomenology of the ayahuasca experience* (Shanon, 2002), we use anecdotal evidence to support our theories and hypotheses.

The GAP dataset is the largest cross-sectional study of ayahuasca drinkers undertaken to date, involving 10,836 people from more than 50 countries and a variety of different traditions, including syncretic religious, traditional ethnobotanical and the so called “neo-shamanic” Western settings. The study utilized an online self-report methodology to collect detailed quantitative and qualitative data. Survey respondents were required to be at least 18 years of age and to have used ayahuasca on at least one occasion. Due to the hidden nature of the ayahuasca drinking population in many countries (where this practice is either prohibited or where its legal status remains unclear), a non-random sampling method was chosen. This enabled the recruitment of a very large number of respondents (*n* = 10,836) that had consumed ayahuasca in traditional, religious and non-traditional settings in more than 50 countries. Survey participation was promoted *via* websites and email invitations from relevant organizations, ayahuasca retreat centers, and ayahuasca churches, online groups and forums, *via* Facebook, and flyers at conferences and events. No financial incentives were offered. Data was cross-checked to remove suspected duplicate responses, while data from partially completed surveys was retained. The study was approved by the University of Melbourne Human Research Ethics Committee (HREC number 1545143.3).

Over 75% of respondents provided some qualitative data relating to their experiences. We note that there is significant variation among GAP respondents in the context of consumption and number of times they have drunk ayahuasca (range 1–5,000). Around 8% of the sample had drunk ayahuasca on a single occasion, 12% two or three times, 19% 4–10 times, 8% 11–20 times, 11% 21–50 times, 15% 51–200 times, 13% over 200 times, and 13% over 500 times. No data was collected about the doses of ayahuasca consumed.

Our previously published work regarding the influence of context on acute and longer-term outcomes identified minimal variation across traditional, church, and neo-shamanic contexts, suggesting common psychotherapeutic processes (Perkins et al., 2022). Similarly, the number of times ayahuasca had been consumed has some influence on the magnitude, but not type of outcomes being attained (Perkins et al., 2022).

## Evidence of therapeutic effects

### Animal and human study evidence

Although still evolving, there is growing scientific evidence indicating that DMT-harmala alkaloid preparations such as ayahuasca may have potential therapeutic utility in the treatment of several psychiatric disorders. Non-clinical and clinical studies, including a small number of double-blind randomized controlled trials have reported evidence of anti-depressive effects (dos Santos et al., 2016b; Osório et al., 2016; Santos et al., 2018; de Almeida et al., 2019; Galvão-Coelho et al., 2021; Sarris et al., 2021), anti-addictive effects (Bouso and Riba, 2014; Nunes et al., 2016; Berlowitz et al., 2019; Perkins et al., 2021a), and anxiolytic effects (Hilber and Chapillon, 2005; Santos et al., 2007; dos Santos et al., 2016b,^2021^). Other articles have hypothesized that ayahuasca consumption may provide therapeutic relief in relation to Post-Traumatic Stress Disorder (PTSD) (Nielson and Megler, 2014), with data suggesting its benefit in suicidality (Zeifman et al., 2019), grief (González et al., 2017, 2020) eating disorders (Lafrance et al., 2017; Renelli et al., 2020); borderline personality disorder (Domínguez-Clavé et al., 2019); and Parkinson’s disease (Serrano-Dueñas et al., 2001; Schwarz et al., 2003).

Sanches et al. (2016) evaluated 17 participants suffering from depression in an in-patient psychiatric unit. Significant reductions in depression were found after a single dose of ayahuasca, maintained at 21 days. Cohen’s *d* was largest at day 7 of follow-up, at 1.83. In a secondary analysis, Zeifman et al. (2019) demonstrated reduced suicidality in this population, with Hedges’s *g* = 1.75 at day 21. Although these studies build on previous results by Osório et al. (2015), there are number of methodological limitation associated with research unto ayahuasca that need to be taken into account. Most studies are open label, and the impressive outcomes reported by authors should be interpreted with caution (Kavenská and Simonová, 2015; Osório et al., 2015; Barbosa et al., 2016; Uthaug et al., 2018; Zeifman et al., 2021). Despite the large effect sizes documented by Zeifman et al. (2019), significant baseline variability in suicidality limits the reliability of these results. Various cofounding variables also limit the validity of results, such as community support in retreats or church settings and associated treatments such as psychotherapy, not to mention significant issues in blinding psychedelic research (Aday et al., 2021).

A systematic review of 28 human ayahuasca studies reported that acute administration was well-tolerated and that neither acute nor long-term use was associated with increased psychopathology or cognitive deficits, but was associated with enhanced mood and cognition, increased spirituality, and reduced impulsivity (dos Santos et al., 2016a). While ayahuasca shows promise for a number of indications, study limitations such as small sample sizes and expectancy bias, which are prevalent throughout psychedelic research, should also be noted and thus further research is needed.

### Psychological and wellbeing effects

Observational and qualitative studies investigating ayahuasca’s therapeutic effects have identified improvements in some psychological skills or traits such as decentring (Franquesa et al., 2018; Domínguez-Clavé et al., 2019); certain mindfulness capabilities (acceptance, non-judgmental and non-reactive processing, and improved observation) (Thomas et al., 2013; Soler et al., 2016; Uthaug et al., 2018); cognitive flexibility (Stemme et al., 2008; Murphy-Beiner and Soar, 2020); and emotional regulation (Domínguez-Clavé et al., 2019).

Broader psychological and wellbeing benefits have also been identified among those drinking ayahuasca. These include increased confidence, optimism, independence, and positive mood (Barbosa et al., 2009), higher levels of self-transcendence and lower harm avoidance (Bouso et al., 2012), increased satisfaction with life (Uthaug et al., 2018) as well as increased openness to therapeutic interventions and improvements in both anxiety and depression (Perkins et al., 2021a). Beneficial effects have also been reported in relation to interpersonal relationships, sense of self, creativity, somatic perception, sense of connection, substance use, and other health behaviors (Thomas et al., 2013; Lafrance et al., 2017; Bathje et al., 2021; Perkins et al., 2021a).

Additional benefit may also be associated with the reported modulation of some personality traits after ayahuasca consumption including increased agreeableness, openness to experience, and extraversion, and reduced neuroticism (Netzband et al., 2020; Weiss et al., 2021) and negative emotionality (Mendes Rocha et al., 2021).

### Proposed neurobiological mechanisms

A range of neurobiological mechanisms have been proposed to contribute to the acute and longer-term therapeutic effects of DMT-harmaloid preparations. These appear to derive from combined effects of DMT (N, N- Dimethyltryptamine related) and the three primary harmala alkaloids (harmine, harmaline, and tetrahydroharmine) (Ruffell et al., 2020). Some of these pathways are shared with other classic psychedelics such as psilocybin and LSD, including the activation of serotonergic, dopaminergic and glutamatergic pathways *via* serotonin 5-HT_2A_ agonism (Carhart-Harris and Nutt, 2017), as well as the ability to induce neurogenesis (Ly et al., 2018), while others are specific to the activation of sigma-1 by DMT (Fontanilla et al., 2009), and that of the harmala alkaloids (Aricioglu-Kartal et al., 2003; Riba et al., 2003; Brierley and Davidson, 2012; Owaisat et al., 2012).

Key neurological processes proposed to be associated with ayahuasca’s therapeutic effects include decreased connectivity in the default mode network (DMN) (Palhano-Fontes et al., 2015; Pasquini et al., 2020), increased neurogenesis and neuroplasticity (Ly et al., 2018), serotonergic and MAOI effects and decreased pro-inflammatory cytokines (dos Santos et al., 2016c), reduced amygdala and insula reactivity (Sanches et al., 2016; Muttoni et al., 2019), as well as modulation of brain regions associated with interoception, emotional processing and volition (Riba et al., 2006; Pasquini et al., 2020).

From a brain network perspective, it is suggested that ayahuasca temporarily modifies the ordinary flow of information within the brain by disrupting usual neural hierarchies (reducing high order cognitive control and facilitating bottom-up information flow), thus facilitating inner exploration and new perspectives on reality *via* the relaxing and revision of existing beliefs (McKenna and Riba, 2015; Carhart-Harris and Friston, 2019). It is also proposed that psychedelics may induce a unique window of adult neuroplasticity (Ly et al., 2018). Interestingly, this period of plasticity shows similarities to that of the adolescent neurodevelopment phase (Lepow et al., 2021).

This wide range of neurobiological effects is likely to facilitate multifaceted psychotherapeutic processes and provides an underpinning rational for the proposed transdiagnostic application of ayahuasca in the treatment of mood and anxiety disorders, addiction, and trauma (see Table 1).

**TABLE 1:** Association between ayahuasca induced neurobiological processes and dysfunctions across psychiatric disorders.

Evidence of modulation by ayahuasca	Role in disorder			
	Depression	Anxiety	Addiction	Trauma
DMN activity (Palhano-Fontes et al., 2015)	Hyperactivated and hyperconnected (Whitfield-Gabrieli and Ford, 2012)	Hypo-connectivity with the affective network and decoupling with the ECN (Xu et al., 2019)	Altered connectivity and emotional processing (Zhang and Volkow, 2019)	Altered resting-state functional connectivity associated with PTSD symptoms (Lanius et al., 2020)
Neuroplasticity (Ly et al., 2018)	Impaired (Price and Duman, 2020)	Impaired (Krystal et al., 2009)	Impaired (Sampedro-Piquero et al., 2019)	Impaired (Kolassa and Elbert, 2007)
Amygdala (Muttoni et al., 2019)	Abnormal functioning (Peluso et al., 2009; Ramasubbu et al., 2014)	Hyper-reactive (Shah et al., 2009; Alvarez et al., 2015)	Role in relapse (See et al., 2003)	Hyperactivity (Bruce et al., 2012)
Right insula (Sanches et al., 2016)	Hypo-active (Wiebking et al., 2015)	Hyper-reactive (Shah et al., 2009; Alvarez et al., 2015)	Dysfunction (Paulus et al., 2013)	Role in PTSD resilience (Jeong et al., 2019)
Interoception (Riba et al., 2006; Wang et al., 2019)	Impaired (Wiebking et al., 2015; Eggart et al., 2019)	Heartbeat hyper-sensitivity (Domschke et al., 2010)	Impaired detection and processing (Paulus et al., 2013)	Impaired (Schaan et al., 2019; Reinhardt et al., 2020)

### Psychotherapeutic processes

While research relating to the neurobiological processes underpinning therapeutic benefits reported with psychedelics compounds is relatively recent, the key role of associated psychotherapeutic processes has been widely recognized since the commencement of psychedelic research in the middle of last century (Leary et al., 1963; Bonny and Pahnke, 1972). A recent systematic review reported several types of psychotherapeutic processes across studies utilizing psilocybin, LSD, ayahuasca, and MDMA in the treatment of various psychiatric disorders. These included an expended emotional spectrum (from bliss and love to anger and terror) (Breeksema et al., 2020) and altered self-perception (increased self-efficacy, reduced self-criticism), increased feelings of connectedness (internally, externally, and with the world/nature), transcendental experiences (mystical, religious, or spiritual) and the gaining of insights (into one’s self, their disorder/s and its origin, interpersonal dynamics).

## A model of psychotherapeutic process associated with ayahuasca consumption

By drawing from the existing literature and combining this information with real-world qualitative data from the GAP study we have developed a more comprehensive understanding of the psychotherapeutic processes associated with ayahuasca consumption, to better inform potential clinical applications. Our model of the psychotherapeutic elements of the ayahuasca experience is outlined below. The five key elements in this framework, discussed further below, are:

1. Somatic effects
2. Introspection and emotional processing
3. Increased self-connection
4. Increase spiritual connection and awareness
5. Gaining of insights and new perspectives

In practice, these elements are highly interconnected, and occur in the context of facilitatory neurobiological effects to achieve beneficial mental health and wellbeing outcomes (Barbosa et al., 2009). However, while these processes typically occur to some degree for most individuals drinking ayahuasca, the relative emphasis will vary and they will not all be present for all those consuming the brew, with significant unpredictability also noted in the literature (Wolff et al., 2019). Our model aims to provide a framework to guide the optimization of clinical treatment models being used with ayahuasca inspired drugs, and well as informing therapists as to what is likely to occur in the sessions and assist them in working with these common features. It should be noted that our trials are to use whole plant material that have undergone lyophilization. We are aware of other groups who are working toward using synthetic DMT and harmala alkaloids (Reconnect Foundation, 2022). Although similarities in the subjective experience will no doubt exist between synesthetic, lyophilizate, and traditional preparations, synthetic forms will lack additional, currently unknown substances often found in plant materials that may well be involved in the biochemical and subjective effects. The processes outlined in this article are based on the experiences induced by ingestion of whole plant material containing DMT and harmala alkaloids. As both Casey et al. (2017) and King et al. (2017) highlight when describing cannabis, plants appear to have evolved with a complex balance of constituents, and therefore processes may differ when synthetic preparations are utilized.

We also note that there are non-pharmacologically induced factors that have been identified as contributing to mental health outcomes associated with ayahuasca consumption. These include drinkers experience of support and safety, the quality of preparation support provided, complementary therapeutic activities, and social strong connections formed in the ayahuasca drinking setting (Perkins et al., 2022). Other factors such as an individuals’ state of mind, or “set,” when consuming ayahuasca, and constituent levels in the brew itself, are also likely to influence their acute experience.

The processes we describe are consistent with those identified in several small prior qualitative studies. In a study of 14 individuals with substance use disorder and 15 therapists utilizing ayahuasca in treatment, Loizaga-Velder and Pazzi (2014a) note four inter-related psychotherapeutic processes: body oriented, transpersonal, insight oriented cognitive, emotional/social. Although these overlap with our model in some respects, notable differences include a narrower interpretation of bodily effects emphasizing enhanced awareness primarily *via* purging, as well as anti-craving and detox effects; the absence of a concept of, and process of connection to, a core Self; and the insight oriented cognitive aspect being concerned primarily with individual psychodynamics and dysfunction, rather broader life direction and life purpose insights or other new perspectives including health behaviors (emphasized in our framework).

Another study of ayahuasca use with 16 individuals with an eating disorder reported psychotherapeutic processes that included the processing of painful feelings and memories, internalization of self-love and acceptance, and spiritual elements of healing (Renelli et al., 2020). A physical/somatic component was not included, but this was mentioned in a related study with the same patient group in the form of strong physical acute effects, and for some, changed understanding or experience of their physical bodies (Lafrance et al., 2017). Finally, a study of regular participants in the Santo Daime ayahuasca church identified psychotherapeutic processes involving insights into individuals’ physical, psychological, emotional, and spiritual health resulting in positive modifications in values and behavior (in particular acceptance and forgiveness, empathy and gratitude), as well as positive dietary and substance use changes, that later of which we consider an outcome (Villaescusa, 2002).

It is noteworthy that although the elements of our proposed psychotherapeutic model have some relevance to other classical psychedelics such as LSD and psilocybin (when used in therapeutic settings), we argue that there are important differences in the case of ayahuasca. Unlike LSD and psilocybin, ayahuasca frequently contains a central somatic element, which is thought to be important in the therapeutic process (Shanon, 2002; Wolff et al., 2019). This can include the well documented purge, which can even modulate emotional and visual experiences and is suggested to be therapeutic in nature (Shanon, 2002; Wolff et al., 2019). Similarly, ayahuasca drinkers have been reported to have more positive and persistent mystical experiences, compared with users of other psychedelics (Davis et al., 2019) and report levels of self-insights and significant life changes beyond those identified with other psychedelics (Perkins et al., 2022).

**FIGURE 1:**
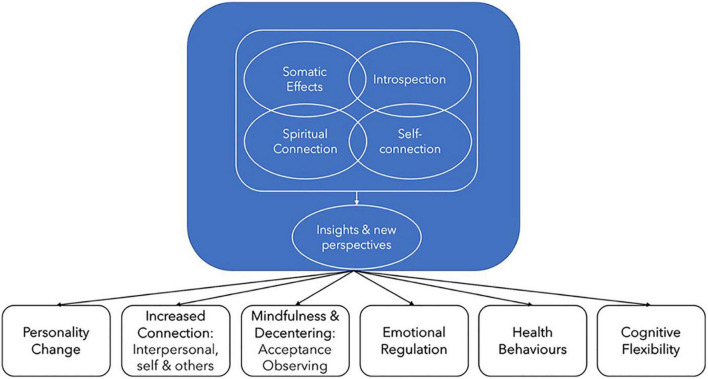
Ayahuasca psychotherapeutic processes and outcomes underpinning improvements in mental health and wellbeing.

As per Figure 1, the first four of these elements are interconnected and together support the gaining of new insights and perspectives, with all these processes then contributing to a range of outcomes that may support enhanced wellbeing and mental health. We believe a therapeutic framework that can connect with and support these psychotherapeutic processes will maximize the likelihood of therapeutic benefit.

### Somatic effects

The acute ayahuasca experience is characterized by strongly enhanced somatic and kinesthetic awareness and interoception (Shanon, 2002; Espinoza, 2014; Kaufman, 2015), with such effects present to a far greater degree than with other psychedelic substances. Somatic effects, both positive and negative, are often strongly associated with other psychotherapeutic processes, and are suggested by Shanon (2014), p. 62 to be “the most basic psychotherapeutic and healing aspect of the ayahuasca experience” (Shanon, 2002, 2014; Espinoza, 2014; Kaufman, 2015).

Somatic effects typically commence with the first perception of the brew being experienced physically by users as an “energetic force, sometimes relating to the plant energy, moving into and taking over the body or nervous system” (Shanon, 2002). Drinkers frequently experience specific healings from ayahuasca somatically and report precise awareness of how traumas and emotional issues are “stored” in and “released” from their bodies.

*“I felt a huge quantity of negative energy coming out of my body*… *and I thought I was dying. She [ayahuasca] spoke to me, she showed me traumatic situations from my childhood and told me that all this negative energy I had in my body was all the residues from those childhood events. After that, I felt like the burden I was carrying my whole life was gone, like someone just took it away”* (GAP Study).

*“I also experienced [ayahuasca] remove a range of emotional, physical and sexual traumas I have experienced. It went thru my veins and body and removed the pain. I released a lot of trauma and negative energy”* (GAP Study).

Throughout the acute experience, drinkers typically have a sense of an altered bodily state, sometimes perceiving something “foreign” having a grasp of their bodies and where physical effects can be of cognitive significance and be integrated with spiritual experiences (Shanon, 2002; Breeksema et al., 2020). Proprioceptive senses are strongly enhanced and frequently include unusually fine degrees of sensitivity, control, or awareness of body parts, often connected with a sense of self-healing, or improved physical health (Shanon, 2002, 2014).

*“I was shown my lungs at a microscopic level and how they work exchanging Oxygen and carbon dioxide and reminded of a broken promise not to smoke again*… *a voice (either mine or something else’s) said ‘you’ve got one more chance’—I have never smoked tobacco in any way since that ceremony 2 years ago.”*

More broadly, the acute experience can involve intense bodily sensations, both positive and negative, such as feeling pure love or embodied traumas and emotional issues, semi-voluntary physical movements typically with a feeling of emotional release, as well as nausea and vomiting. Ayahuasca drinkers often experience what is known as “the purge” with the brew being referred to as “la purga” by some traditional practitioners (Domínguez-Clavé et al., 2016). This is largely due to the alteration in enzymes in the stomach caused by the harmala alkaloids as well as the effect on the serotonin receptors in the gut by DMT (Gershon, 2004).

Such vomiting is traditionally considered a positive effect and is often reported by ayahuasca drinkers to provide immense emotional and psychological relief, connection with usually inaccessible emotions, and feelings of joy and personal transformation, in some cases life-long (Shanon, 2002, 2014; Gearin, 2015; Lafrance et al., 2017; Rush et al., 2021).

Neurological studies of ayahuasca consumption have identified significant activation of the neural systems involved with interoception and emotional processing, particularly frontal and paralimbic areas and the anterior insula (right hemisphere), all of which have been associated with somatic awareness and heightened conscious perception of emotional and affective states (Riba et al., 2006; Wang et al., 2019). The importance of ayahuasca’s somatic aspect is also highlighted by a growing body of literature pointing to the neurological integration of interoceptive and emotional activity (Seth and Friston, 2016; Strigo and Craig, 2016; Tsakiris and Critchley, 2016).

In addition to heightened somatic awareness in the acute experience, there is also evidence of reduced bodily dissociation from longitudinal and qualitative studies, a notion also conveyed by ayahuasca drinkers (Espinoza, 2014; Kaufman, 2015).

*“I feel that my body is so much more present in my life now than before taking ayahuasca. It’s as if I’ve been stuck in my thinking my whole life, and now that’s different*” (GAP Study).

Similarly, in a qualitative study of 41 ayahuasca drinkers, Bathje et al. (2021) report participants commonly feeling different in their bodies after ayahuasca, with changes such as experiencing less bodily chronic stress, feeling more in tune with bodily sensations, relief from tics or muscle twitches, and feeling more energy, lightness, or a release or psychical/energetic blockages.

Such enhanced somatic awareness that may be achieved with ayahuasca can be therapeutically valuable in allowing connection with and processing of deep emotional material, potentially leading to better differentiation of emotions associated with bodily changes and improved emotional perception and regulation (Van der Kolk, 2014; Schaan et al., 2019).

*“I was holding on to trauma and fear. Ayahuasca showed me how to let go of it and gave me a new perspective to see myself through. This experience reminded me that I had forgotten how it feels to be relaxed in my body and Ayahuasca showed me the way back to this resting state”* (GAP Study).

*“Ayahuasca drew my awareness from my head back down into my body. I had cut myself off from feeling much in my body after bad experiences in my early teens and it helped to reconnect me”* (GAP Study).

Such effects are consistent with the new wave of somatic-based psychological therapies (Davis, 2021) and are of relevance for various psychiatric disorders, including addiction (Paulus et al., 2013), depression (Wiebking et al., 2015), PTSD (Reinhardt et al., 2020), and childhood trauma (Schaan et al., 2019), where impaired interoceptive processing and anterior insula function, have been identified.

### Introspection and emotional processing

The acute ayahuasca experience typically results in profound self-analysis in the form of visions and dreamlike sequences, an evocation of intense emotions, and the recollection and reprocessing of significant autobiographical material, including early life events, unresolved traumas, important relationships, experiences, or people (Shanon, 2002; Echenhofer, 2011; Perkins and Sarris, 2021).

The level of mental clarity and emotional awareness is enhanced, enabling better recognition of dysfunctional thoughts and emotions, and maladaptive behavioral, emotional, and cognitive patterns, which can then be reframed, restructured and integrated in an accelerated psychotherapeutic process (Frecska et al., 2016; Franquesa et al., 2018; Argento et al., 2019; Renelli et al., 2020; Ruffell et al., 2021; Scheidegger, 2021).

*“Ayahuasca has made me see why I think, feel, etc. as I do. It’s helped me see and break negative patterns in my life, overcome my fears, and give me a deep sense of peace. It was like 20 years of therapy in my first weekend”* (GAP Study).

*“I carried a tremendous amount of suppressed grief from a childhood of abuse, followed by an abusive first marriage. Drinking Ayahuasca has helped me to experience a ‘life review,’ pulling up suppressed painful memories, looking at them, processing them, and receiving, what I believe was crucial insight”* (GAP Study).

Consistent with such effects, a study of ayahuasca drinkers in Brazil identified quickened thought processes, exceptional understanding, “Aha!” experiences, and feelings of clarity to be reported by around 90% of participants (Bresnick and Levin, 2006). These processes can involve important reflections on the aetiology of mental health conditions, poor health behaviors, addictions, and destructive relationship patterns (Loizaga-Velder and Verres, 2014b; Franquesa et al., 2018; Maia et al., 2020). At times this can be emotionally confronting as participants feel their usual defenses peeled away and are confronted with denied or unpleasant aspects of themselves and their innermost fears (Loizaga-Velder and Verres, 2014b; Franquesa et al., 2018; Maia et al., 2020). As Shanon (2002) describes, “One is cruelly confronted with one’s self, and one finds oneself having no other option but to address issues that are often neither easy nor pleasant to handle.” However, such difficult experiences are also commonly acknowledged by drinkers as useful learning or therapeutic experiences (Shanon, 2002; Bresnick and Levin, 2006; Kjellgren et al., 2009; Loizaga-Velder and Verres, 2014b; Franquesa et al., 2018; Maia et al., 2020).

*“My first experience was profound love and recognizing the lack of love from my childhood aka abuse, etc. My second experience went more deeply into the abuse from my childhood and was very hard, painful and scary, but necessary, and there was a sense of relief after”* (GAP Study).

Neurologically this process appears to be facilitated through the activation of neural systems associated with emotional processing and memory, providing access to deeply stored emotional material, while at the same time stimulating higher cortical areas, enabling processing and reconceptualizing of meaningful events and cognitive-emotional integration (Riba et al., 2006; Nielson and Megler, 2014). The proposed disruption of neural hierarchies, discussed earlier, is also likely to support this emotional reprocessing *via* the relaxing and revision of existing beliefs.

### Increased self-connection, value, and love

As has been noted with other psychedelics, the ayahuasca experience is commonly reported to result in a shift in self-perception. However, with ayahuasca this goes beyond simply a reframing of previous self-perceptions, to include a renewed connection to what can be described as an authentic, wise, and compassionate core “Self” as well as a renewed sense of self-love and value (Fericgla, 2018; Renelli et al., 2020).

This “Self” concept is consistent with that described by Schwartz as a “spacious essence in each person” that, when accessed spontaneously, manifests leadership qualities including acceptance, calmness, clarity, compassion, perspective, and kindness, and has parallels in several Eastern spiritual traditions, and some humanistic psychology approaches (Schwartz, 2013; Sweezy et al., 2013; Anderson et al., 2017).

The connection with this Self during ayahuasca experiences appears central in enabling emotional openness, introspection and associated challenging experiences to progress toward acceptance and emotional release. Of particular importance appears to be the harnessing of compassion and understanding and the removal of deeply held judgments about individuals themselves, their behaviors, and other people or events.

*“Showed the darkest parts in me and the worst of my behavior and gave me the opportunity to forgive myself which helped me forgive those who have wronged me”* (GAP Study).

After drinking ayahuasca, participants have been found to experience a greater sense of self-awareness, self-acceptance, and presence in themselves, as well as enhanced self-love, leading to greater empathy and improved interpersonal relationships (Kjellgren et al., 2009; Soler et al., 2016; Renelli et al., 2020). Such renewed Self-connection is evident in increased generalized self-efficacy, personal authenticity (Perkins et al., 2022). These reported improvements in psychological wellbeing have been found to be strongly predictive of better current mental health (Perkins et al., 2022).

The increased Self-connection experienced with ayahuasca may also be central to reported increases in “decentring,” the meta-cognitive ability to take a detached view of thoughts and emotions (Fresco et al., 2007; Soler et al., 2016). This ability has been postulated as a trans-diagnostic index in psychopathology (Soler et al., 2016).

Several studies have also reported profound shifts in drinkers’ self-acceptance and self-love to be critical in catalyzing mental health and addiction outcomes (Thomas et al., 2013; Argento et al., 2019; Renelli et al., 2020), which was also reported by GAP participants.

*“Ayahuasca helped me recognize my self-destructive ways using drugs and alcohol. The lessons of self-love and being true to oneself showed me that I did not want to poison my body, mind and spirit anymore with these substances”* (GAP Study).

*“Greatly increased self-love. I now care a lot more about my well-being like you would for a loved one. Greatly increased emotional availability. Feel more in touch with my inner self. Motivated to make changes in life motivated by self-love”* (GAP Study).

Data from the GAP study confirms the central place of this self-concept, with “higher” or “true” self this being the most commonly selected interpretation (>70%) of the source of information or insights gained during the acute experience (Perkins et al., 2023).

### Increased spiritual connection and awareness

The strength of the acute subjective spiritual experience has been identified as a strong predictor of therapeutic outcomes for individuals taking classical psychedelics, including ayahuasca (Russ et al., 2019; Perkins et al., 2021a,^2022^). However, when compared with psilocybin or LSD, ayahuasca drinkers have been reported to have more positive and enduring mystical experiences with more lasting impacts on life satisfaction, social relationships, spiritual awareness, mood, and behavior (Davis et al., 2019).

The ayahuasca experience is identified as among the top five most spiritually significant life experiences by around 70% of GAP study respondents, with over three quarters reporting insights or new perspectives regarding “a sense of sacredness, higher power or divine in the world,” and almost 90% indicating this has had a very positive effect on their life (Perkins et al., 2023). Other commonly reported aspects of such enhanced spiritual connection, by more than half of drinkers, includes a sense of connection and kinship with the natural world, enduring connection with a plant spirit or intelligence, and a sense of interconnection between all things/events (Perkins et al., 2023), which have also been reported in other research (Shanon, 2002; Harris and Gurel, 2012).

It appears that the deepened connection to nature reported is part of a broader increase in connectedness to Self, others, world/universe and a spiritual principle that has been associated with psychedelics such as psilocybin (Carhart-Harris et al., 2017; Watts et al., 2017). These peak experiences and spiritual insights have been proposed to be therapeutically useful in supporting a new orientation to life in general (Kjellgren et al., 2009), as well as in catalyzing spiritual elements of healing from addictions and other psychiatric conditions (Liester and Prickett, 2012; Loizaga-Velder and Pazzi, 2014a; Renelli et al., 2020).

A further common and unique aspect of the ayahuasca spiritual experience is the perception of receiving pure or divine love from plants, spirits, or other divine sources, which individuals can find profoundly transformative.

*“I had previously been suicidal but the intense love I felt from the plants and the understanding of life that they gave me made it impossible for me to ever seriously consider this again”* (GAP Study).

*“As a previous agnostic/atheist, I experienced infinite love from the divine/God, how my true self/consciousness is linked to everything and everyone and that my soul is eternal. My depression was cured. My crushing anxiety was cured*” (GAP Study).

New understandings and perspectives on death and dying are also regularly reported and can sometimes involve perceived near death experiences. Such experiences with psychedelics have been reported to confer long-term positive changes in well-being (Timmermann et al., 2018) and may support change in unhealthy behaviors or psychological symptoms, and reduced anxiety of death among individuals with serious illnesses (Timmermann et al., 2018; Maia et al., 2020).

*“I experienced the most intense level of fear I have ever experienced. I was out of my body and propelled into oblivion and faced death. After experiencing this level of fear, I have lost most of my anxiety in real life”* (GAP Study).

*“I’ve visited death twice during ceremony, the first time being difficult because of resistance and confusion, The second being beautiful because of acceptance and surrender. Both experiences, in their own way, taught me that death is not something to fear. I have the greatest and most humble respect for Divine Creation”* (GAP Study).

Analysis of longitudinal data of ayahuasca naive drinkers has also identified increased levels of intrinsic spirituality (Bouso et al., 2012; Perkins et al., 2022). The use of a psychospiritual therapeutic support framework has been recommended based on data showing that spiritual/religious counseling provided with ayahuasca drinking (prior to/after the acute experience) is associated with a greater number of personal insights, reduced integration difficulties, greater perceived improvements in psychological wellbeing, and better current mental health status (Perkins et al., 2022). This is understandable given the crucial place spiritual experiences often have in transforming mental health symptoms and facilitating wellbeing and life meaning for ayahuasca drinkers.

### Insights and new perspectives

Closely connected with each of the areas above is the gaining of new insights and perspectives relating to an individual’s past, present and future, which can then have profound meaning and therapeutic value (Kjellgren et al., 2009; Frecska et al., 2016). The gaining of these insights and perspectives (primarily during the acute experience) can be seen as offering the tangible potential for life change beyond simply introspection and emotional processing. Such insights during the ayahuasca experience have been found to be associated with improved psychological wellbeing, reduced alcohol and drug use, and reduced depression and anxiety symptoms among those with a prior diagnosis (Perkins et al., 2021a,^2022^; Sarris et al., 2021).

The most common personal insights reported by drinkers in the GAP study include new understandings of their personality, new understandings about family and personal relationships, realizations about ethics, morals and their own conduct, revelations about their life purpose and direction, new understandings of childhood events, patterns/dynamics in intimate relationships, and new understandings of their body function and care, all of which were reported by over 50% of participants. Moreover, almost 80% of participants who had drunk ayahuasca on only one occasion reported at least three such personal insight (Perkins et al., 2023). Other research has reported similar interpersonal, psychodynamic, and life purpose themes in drinkers’ insights (Loizaga-Velder and Verres, 2014b; Kavenská and Simonová, 2015; Wolff et al., 2019), as well as insights relating to health and bodily functioning and care (Shanon, 2002), and other new perspectives such as personal or creative interests (Shanon, 2002; Kjellgren et al., 2009; Kavenská and Simonová, 2015; Wolff et al., 2019).

There is evidence that these insights and new perspectives can contribute to meaningful life changes (Bouso et al., 2012; Franquesa et al., 2018). Such changes commonly attributed to ayahuasca by GAP participants include the healing of longstanding personal rifts/conflicts, reductions in drug and alcohol use, and changing career or commencing study in a new area, each of which was reported by 30–50% of study participants (Perkins et al., 2023). Insights and life changes were also correlated, with for example, those reporting insights about their life purpose and direction being more likely to report subsequent vocational changes (Perkins et al., 2023).

New insights and perspectives relating to alcohol and other drug use, diet, and other health behaviors is a key area that may provide ongoing therapeutic benefit (Loizaga-Velder, 2013; Maia et al., 2020). Around 50% of GAP respondents report taking care of their physical health and eating a healthier diet “much more” than previously, with such responses more likely among drinkers also reporting insights about their body function and care (Perkins et al., 2023).

Post ayahuasca, new perspectives on substance use described in terms such as “losing interest,” the “taste for alcohol,” or no longer liking how it felt in their bodies are commonly reported, for example:

*“I never considered myself to have an alcohol problem. About 3 weeks after the experience, I tried a sip of beer and it made me feel terrible. I realized that I never enjoyed the effect of alcohol in my body, and that I would never drink it again. I give ayahuasca credit for both opening me to the reality that alcohol was bad for my body, and that I had the strength to make the choice to stop drinking it.”*

*“I feel more embodied and comfortable in my body. I find I cannot drink any more than a glass of wine or a beer and even then, I’ll lose interest halfway through. More than a glass and I feel queasy and toxic. I used to be heavily dependent on alcohol for stress relief (every night up to a bottle of wine on my own).”*

### Integrated effects

In practice, the proposed psychotherapeutic processes will usually be interconnected and mutually supportive, with for example introspection and emotional processing being facilitated by the connection to Self, somatic awareness and in some instances the spiritual connection. Such interconnected healing effects are also apparent in the healing descriptions of drinkers.

*“After engaging with the medicine, I have a deeper sense of myself, this physical body, as well as the spirit (ME, both the individual and universal selves). How I am connected, interconnected, and interrelated to all beings. I can see that my disease is a manifestation of historical trauma on my mother’s side, as well as my own childhood/sexual traumas*… *and that those energies/experiences were lodged deep within my body, leading to disease”* (GAP Study).

*“It was life changing. It has helped me to release my childhood traumas, come to a place of love and acceptance and released a number of emotional blockages in my body, after which I now have the ability to feel energy of myself and others”* (GAP Study).

*“Drinking ayahuasca was life changing in that it made me go inside. It changed my focus to my inner world rather than just reacting to my environment. It helped me understand myself and others much more than I ever did before, which improved my relationships. It also changed my relationship with my body, my food choices, it made me lose interest in alcohol”* (GAP Study).

## Implications for therapeutic application

Contemporary therapeutic approaches to the use of psychedelic compounds have differed somewhat depending on the substance and conditions being treated as well as the choice of an overarching or primary therapeutic framework (e.g., acceptance and commitment therapy -ACT, mindfulness-based cognitive therapy—MBCT, Internal Family Systems Therapy—IFS, or non-specific approaches). However, they all share a common structure based on the provision of psychotherapeutic support for participants: to prepare for the drug administration session, during the drug administration session, and to assist with integration in the days or weeks following drug administration (Schenberg, 2018). Our model of psychotherapeutic processes associated with the ayahuasca experience provides unique and directly relevant insights to inform potential DMT-harmala alkaloid clinical treatment models at each of these phases.

Given the central place of somatic effects in ayahuasca’s psychotherapeutic outcomes and the apparent enhanced somatic awareness window created (effects not generally seen with other psychedelics), the inclusion of additional somatic processing practices/tools and therapeutic frameworks incorporating a somatic dimension, is likely to be of significant benefit to participants. The inclusion of this focus in preparation and integration will support a deepening of the acute experience [this has been reported for yoga/tai-chi (Perkins et al., 2022)] and more effective post-administration processing of experiences and emotional content. While other psychedelic therapy approaches provide some guidance on incorporating somatic tools such as focused bodywork or breathwork, these are not integrated as a core element of the approach and are dependent on the prior training of the therapist.

The common experience of connection to a core Self, which is a source of wise guidance, compassionate perspective, and leadership, is another novel aspect of the ayahuasca experience, and something that for many individuals with treatment resistant psychiatric disorders may have previously been substantially or entirely absent. The use of therapeutic frameworks that can foster a continued connection to and development of this inner resource *via* a non-pathologizing, participant led approach, is likely to have a range of benefits. These include supporting the effective processing of emotional material revealed during acute experiences, strengthening the individuals’ personal capabilities and Self-leadership, and facilitating enduring therapeutic benefit. As the ayahuasca core Self connection is typically also a central aspect of the introspection and emotional processing, and insights and new perspectives processes, we believe this lens is ideally suited to working with such material therapeutically. In this regard it would also be beneficial for the treatment model to specifically note the wide range of life, personal and vocational changes that can be catalyzed by the ayahuasca experience and highlight the type of support likely required for following through and implementing such changes.

The spiritual process identified in our model is shared by other frameworks and has been identified as important across the classic psychedelics (psilocybin, LSD, DMT) (Breeksema et al., 2020). However, there is evidence of a greater level of depth to this experience with ayahuasca. As such, we suggest that the use of therapeutic frameworks that include a psycho-spiritual element is likely to provide benefits for participants in clinical settings, who unlike traditional users of this brew, typically have no frame of reference in which to prepare for, understand, or integrate the powerful spiritual experiences commonly reported. Consistent with this idea, the use of spiritual or religious counseling across different naturalistic contexts of consumption has been associated with enhanced mental health outcomes (Perkins et al., 2022). In relation to the use of a psycho-spiritual framework, we also note the high level of openness, vulnerability and suggestibility that can be induced by ayahuasca experiences, and hence the ethical need to ensure that such an approach is culturally sensitive, non-doctrinal, and personally empowering (allowing participants to derive their own meaning and not have this imposed by a therapist).

Finally, as mentioned above, we believe our model provides important information regarding the selection of key therapeutic frameworks to inform clinical use. In this regard we see the Internal Family Systems (IFS) therapy approach as a noteworthy candidate. It is an evidence-based approach that has a strong somatic element, is centered around the concept of a core Self involving many similarities to that experienced by individuals using ayahuasca, and with a goal of cultivating connection to this core-Self utilizing a non-pathologizing, participant led approach. Further IFS incorporates a broad psycho-spiritual framework that is compatible with a wide range of personal spiritual or religious beliefs, and in which individuals are viewed as inherently spiritual beings (Schwartz, 1995; Sweezy et al., 2013; Janes et al., 2022). However, we note other therapeutic approaches may also address the psychotherapeutic elements identified in our model, and believe the optimal approach will likely involve an adapted version of one or more of these frameworks with additional supportive somatic and other tools.

### Potential therapeutic challenges

Despite the range of positive effects typically associated with the psychotherapeutic processes outlined, it is also important to note ways in which these may also contribute to therapeutic challenges. The introspection and emotional processing element with ayahuasca, for example, can involve a particularly vivid reconnection with or re-experiencing of prior traumatic experiences, including early life events. This is usually reported to be highly therapeutic, however, for vulnerable individuals or in the absence of appropriate support, such effects may create mental health difficulties or re-traumatization (Neilson and Jeffrey., 2018).

*“It’s opened up old deep wounds, with some harsh uncomfortable truths, which I’m now slowly processing in weekly psychotherapy”* (GAP Study).

Similarly, the strong somatic effects can deconstruct defense mechanisms such as dissociation and diminished interoception that are common among trauma survivors and shield them from experiencing overwhelming emotion (Van der Kolk, 2014), as is described by (Perkins and Sarris, 2021, p. 109):

*“I experienced her (ayahuasca) in Peru. Aya (ayahuasca) removed all the blocks that I had which were preventing me to feel the childhood traumas. It did a great job removing the blocks and because my environment was perfect at that time, I felt like in heaven. I felt one with the universe and I obtained a great physical health. After returning home, life was not so perfect anymore, and I started to feel all the traumas and now there was no block, nothing to stop me to FEEL the pain caused by traumas.”*

Spiritual experiences that include a feeling of imminent death along with extreme fear or panic have been associated with integration difficulties and longer term negative mental health outcomes (dos Santos et al., 2017; Perkins et al., 2021a). Further, the remarkable clarity of insights and new perspectives gained with ayahuasca may in rare cases lead to problematic interpretations such as rigid attitudes, grandiose thinking, ego inflation, narcissism, or “spiritual narcissism” (Gastelumendi, 2010; Fernández and Fábregas, 2014; Loizaga-Velder and Pazzi, 2014a; Fericgla, 2018). In other cases this may involve rushed major decisions such as changing career or ending a relationship, or exaggerated or unusually uninhibited behaviors expressing feelings, psychological wishes, fantasies, or conflicts (Shanon, 2002).

More broadly the profound and multifaceted psychotherapeutic effects can prove a challenge for ayahuasca drinkers to assimilate and integrate and it is not uncommon for individuals to experience some level of emotional or psychological difficulty in the weeks or months following their experiences, with a higher number of such difficulties associated with poorer long term mental health outcomes (Perkins et al., 2021a).

## Discussion

The resurgence in psychedelic research that has occurred over the last decade is often referred to as the “psychedelic renaissance” (Sessa, 2012). Preliminary studies are showing promising results for compounds including MDMA for PTSD (Mithoefer et al., 2018; Mitchell et al., 2021), psilocybin (Carhart-Harris et al., 2021; Gukasyan et al., 2022), and ayahuasca for depression (Palhano-Fontes et al., 2019). As such, there is substantial work underway seeking to understand the neurobiological mechanisms that may facilitate such change (Riba et al., 2002, 2006; Sampedro et al., 2017). At the same time and in recognition of the ability of these substances to induce profound psychological, emotional and spiritual experiences, guidelines for safety (Johnson et al., 2008) and various manualized treatment models are being developed by research groups around the globe providing guidance for those working with these substances in clinical research settings (Mithoefer, 2017; Guss et al., 2020; Watts and Luoma, 2020). The aim of these treatment models is to provide standardized support that will maximize benefits and minimize harms associated with such treatments. However, the development and evaluation of such models remains in its infancy. Further, there has been no manualized approach developed for the potential clinical use of DMT-harmala alkaloid based drugs such as ayahuasca, and the accompanying psychotherapeutic processes are not yet well-defined in the scientific literature (Franquesa et al., 2018).

Our framework serves to clarify and extend the existing knowledge of psychotherapeutic processes associated with the consumption of ayahuasca in a succinct and relevant way. It provides critical information for the potential clinical use of ayahuasca inspired drugs by identifying key areas in which psychotherapeutic treatment models should be congruent with the inherent ayahuasca based therapeutic processes, in order to maximize benefit to patients. In additional to treatment model design, the five elements of our model will also be useful for therapist training by providing a clearer understanding of processes that are likely to emerge in DMT-harmaloid assisted sessions in a clinical setting. We hypothesize that an awareness and understanding of the above will help to catalyze positive therapeutic outcomes. At the time of writing, we are developing training for therapists to work with a DMT-harmaloid concoctions in a clinical setting, with a focus on these five experiences.

Further definition of the powerful psychotherapeutic processes involved with psychedelic compounds, *via* models such as that proposed in this article, also helps to elucidate the integrated psychotherapeutic and neurobiological source of remarkable outcomes being achieved in clinical trials to date. This information provides a valuable perspective from which to reflect on the potential efficacy of a new class of non-hallucinogenic psychedelic-like compounds (Peters and Olson, 2021). In the case of ayahuasca, which we note involves key neurobiological processes proposed for emulation in non-hallucinogenic “psychedelics” (such as 5-HT2A agonism and deactivation of the DMN), we believe our review and model suggest an indispensable role of the acute altered state experience in catalyzing therapeutic processes reported.

### Limitations

There are also certain limitations that should be noted in relation to our review. Although a strength of the GAP dataset is its size and inclusion of ayahuasca drinkers across different contexts of consumption in a range of countries, it has minimal representation of Indigenous ayahuasca drinkers in countries such as Peru, and in Brazil the sample is primarily from the ayahuasca churches., Further, it utilizes a voluntary self-selected sample that may lead to an overrepresentation of participants who are enthusiastic about ayahuasca and motivated to spend time completing the survey, vs. those with neutral or negative experiences. It would be beneficial for future studies to operationalize and evaluate the individual and joint effects of the five key psychotherapeutic processes we propose, with this of particular value in well-controlled clinical contexts.

It should also be noted that although we suggest the experiences outlined in this article are associated with therapeutic outcomes, this relationship has not been proven to be causal. Further research is required to further explore this hypothesis. In addition, the ayahuasca experience is extremely subjective, and although this will typically involve the five processes we propose to some degree, there will also be significant variability in experience both between and within participants (Shanon, 2002; Wolff et al., 2019). It is therefore possible that our model can be further refined as more data is collected through our clinical work.

Finally, while we believe the psychotherapeutic framework described will assist the design of appropriate therapeutic treatment models and training of therapists, and consequently improve patient outcomes, we also note that such potential clinical use of ayahuasca raises other issues relating to appropriate recognition and reciprocal relationships with indigenous communities from whom knowledge of ayahuasca originates.

## Data availability statement

The data analyzed in this study is subject to the following licenses/restrictions: The datasets presented in this article are not readily available because ethics approval and consent signed by participants was for data access by research team members only. Requests to access these datasets should be directed to DP, d.perkins@unimelb.edu.au.

## Ethics statement

The studies involving human participants were reviewed and approved by the University of Melbourne Human Research Ethics Committee (HREC number 1545143.3). The patients/participants provided their written informed consent to participate in this study.

## Author contributions

DP and JS were involved in the GAP data collection. DP, SR, KD, and DPR wrote sections of the manuscript. All authors contributed to the manuscript revision, read, and approved the submitted version.
